# Full-length transcriptome sequencing from multiple tissues of duck, Anas platyrhynchos

**DOI:** 10.1038/s41597-019-0293-1

**Published:** 2019-11-21

**Authors:** ZhongTao Yin, Fan Zhang, Jacqueline Smith, Richard Kuo, Zhuo-Cheng Hou

**Affiliations:** 1 Department of Animal Genetics, Breeding and Reproduction, College of Animal Science and Technology, China Agricultural University, Beijing, 100193 China; 2 The Roslin Institute & R(D)SVS, University of Edinburgh, Easter Bush, Midlothian, EH25 9RG UK

**Keywords:** Sequence annotation, RNA sequencing, Evolutionary biology, RNA splicing

## Abstract

Duck (*Anas platyrhynchos*), one of the most economically important waterfowl, is an ideal model for studying the immune protection mechanism of birds. An incomplete duck reference genome and very limited availability of full-length cDNAs has hindered the identification of alternatively spliced transcripts and slowed down many basic studies in ducks. We applied PacBio Iso-Seq technologies to multiple tissues from duck for use in transcriptome sequencing. We obtained 199,993 full-length transcripts and comprehensively annotated these transcripts. 23,755 lncRNAs were predicted from all identified transcripts and 35,031 alternative splicing events, which divided into 5 models, were accurately predicted from 3,346 genes. Our data constitute a large increase in the known number of both lncRNA, and alternatively spliced transcripts of duck and plays an important role in improving current genome annotation. In addition, the data will be extremely useful for functional studies in other birds.

## Abstract (summary)

**Table:** 

Measurement(s)	FL-cDNA • sequence annotation • Alternate Splicing • transcription profiling assay • sequence_assembly
Technology Type(s)	PacBio Sequel System • bioinformatics analysis • RNA sequencing • genome assembly
Sample Characteristic - Organism	Anas platyrhynchos

Machine-accessible metadata file describing the reported data: 10.6084/m9.figshare.10255526

## Background & Summary

Duck (***Anas platyrhynchos***), one of the most economically important waterfowl, is an ideal model for studying the protection offered by the immune system in birds. Insights have been obtained using various transcriptomic datasets from multiple tissues, developmental times and environmental backgrounds^1–4^. The duck genome reference assembly, released in 2013, used the duck genetic map and the comparative physical map with the aim of completely covering the gene space^5^. The high-quality genome annotations which relied on evidence-based approaches required various transcriptomic datasets. Although short-read sequencing data of duck have accumulated over recent years, full-length (FL) RNAseq datasets are not currently available in domestic ducks, limiting genome annotation and the ability for identifying alternatively spliced genes. In addition, low-quality transcripts assembled from short-read sequencing will reduce the accuracy of annotations^6^.

Alternative splicing (AS) is prevalent in most eukaryotic genomes, and is a mechanism by which an organism can increase its repertoire of proteins and regulate physiological and developmental processes/pathways^7–11^. The diversity and complexity of AS increase the difficulties faced in genetic research. Studies of AS in duck are scarce, and likewise, there is a lack of such information in most birds due to the absence of detailed full-length cDNA data and high-quality genome annotation^12^. The method of Sanger sequencing of full-length cDNA clones has provided a reliable standard for genome annotation projects^13–15^. Recently, this method has been replaced by cheaper short-read technologies. However, the short-reads make it difficult to define the actual combinations of splice-site, increasing false positive AS prediction. The PacBio single-molecule technology can obtain actual sequences for transcript isoforms of each gene without assembly^16–18^. This technology provides more evidence for AS and improves the accuracy of genome annotation^19–21^.

There are few studies of AS in ducks (or other birds)^22^. To begin to address this, we carried out PacBio long-read transcriptome sequencing on multiple tissues from duck. We multiplexed eight tissues to ensure coverage of transcript isoforms and pooled them for subsequent sequencing. We obtained a total of 199,993 full-length transcripts ranging in size from 206 bp to 15,233 bp. The number of transcript sequences annotated to NCBI non-redundant protein sequences (Nr), NCBI nucleotide sequences (Nt) and the UniprotKB database is 127,780, 185,435 and 102,539, respectively. Furthermore, there are 116,503, 82,456 and 97,823 transcripts corresponding to the Kyoto Encyclopedia of Genes and Genomes (KEGG), euKaryotic Ortholog Groups (KOG) and Gene Ontology (GO) databases for providing functional annotations, respectively. In addition, a total of 35,031 AS events were detected in the unigenes, while 23,755 lncRNAs were identified in multiple tissues. Our study provides the first comprehensive datasets describing AS events and lncRNA transcripts in *Anas platyrhynchos*, which will be useful for further AS evolution studies in birds. This data will also serve as an important dataset for genome annotation.

## Method

### Sample collection and RNA preparation

Duck samples (both adult and embryos) were obtained from Pekin Gold Duck Inc. We collected 8 tissues (pectoralis, heart, uterus, ovary, testis, hypothalamus, pituitary and 13 days-old embryo) in order to obtain comprehensive transcript information. Tissue samples were sampled immediately after euthanization, snap-frozen in liquid nitrogen and then stored at −80 °C until RNA extraction. RNA from each tissue was extracted individually (10 μg per tissue) using Trizol reagent (Invitrogen, CA, USA) according to the manufacturer’s instructions. RNA concentration was assessed using a NanoDropTM spectrophotometer (Thermo Fisher Scientific, Waltham, USA), and RNA integrity number (RIN) values were calculated using an Agilent 2100 Bioanalyzer (Agilent Technologies, Santa Clara, USA) (Table 1).

**Table 1:** The purity and completeness of RNA for Iso-seq library.

Sample	Library	Accession IDs	Purity		Completeness (RIN)
			OD260/280	OD260/230	
Pectoralis	Library1	SRX5511971	2.15	1.88	7.9
Hypothalamus			1.83	1.61	8.5
Hypophysis			1.98	1.83	8.2
Uterus			2.04	2.14	8.8
Ovary	Library2	SRX5511972	1.89	1.71	9.2
Testis			2.02	2.13	9.5
Heart			2.02	2.1	8.3
Embryo			2.02	2.2	8.4

### Library construction

We built two iso-seq libraries from 8 tissues, in which pectoralis, uterus, hypothalamus, and pituitary were pooled to make one library, and heart, ovary, testis, and embryo were pooled for the other. Equal amounts of RNA from each tissue were mixed (5 μg per tissue) to construct the iso-seq libraries. Sequencing libraries were generated according to PacBio’s iso-seq sequencing protocol. Briefly, the Clontech SMARTER cDNA synthesis kit with Oligo-dT primers was used to generate first- and second-strand cDNA from polyA mRNA. Size fractionation and selection (<4 kb and >4 kb) were performed using the BluePippin™ Size Selection System (Sage Science, Beverly, MA). Two SMRT bell libraries were constructed with the Pacific Biosciences DNA Template Prep Kit 2.0 and SMRT sequencing was then performed using the Pacific Bioscience Sequel System.

### Full-length sequencing and analysis pipeline

**Fig. 1:**
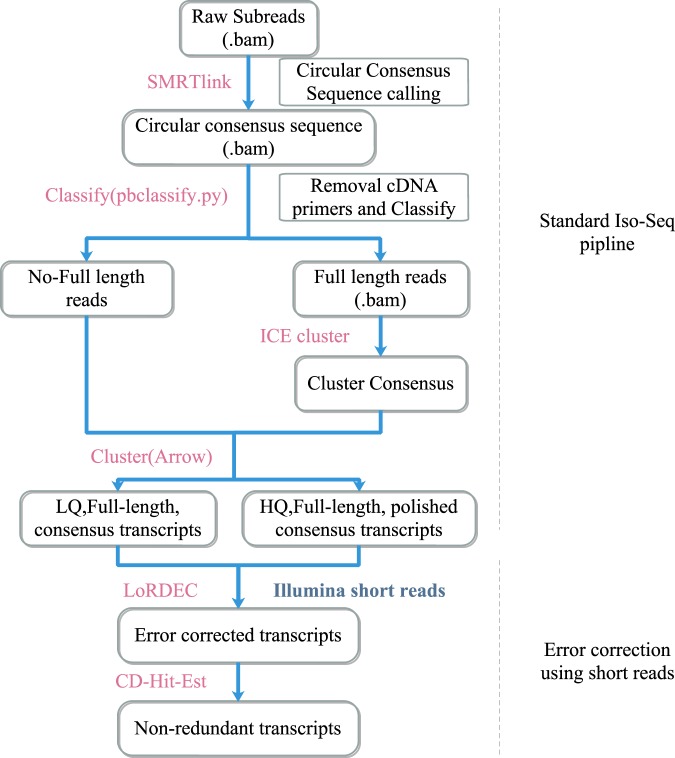
The standard Iso-Seq pipeline for raw data processing. Raw sequence reads from a Pacbio RSII sequencer were processed using SMRTlink. The full-length reads and non-full-length reads were clustered into consensus transcripts using Arrow. All polished reads were corrected with Illumina short-read data using LoRDEC. All sequence data that removed redundant sequences using CD-Hit-Est were carried on to further analysis.

We combined all raw data and performed initial data processing according to the Iso-seq standard pipeline (Fig. 1). The Circular consensus sequence (CCS) was generated from initial data using the SMRTlink (version 5.1) software^16^. The CCS was classified into full-length and non-full length reads according to the 5′and 3′adapters and the poly(A) tail. Reads containing both the 5′ and 3′ primers and having a poly(A) tail signal preceding the 3′ primer were considered to be full-length reads. Iterative Clustering for Error Correction (ICE) was used to find transcript clusters based on the pairwise alignment and reiterative assignment of full-length reads. The cluster consensus reads were polished with non-full length reads to obtain high-quality isoforms using Arrow software(https://downloads.pacbcloud.com/public/software/installers/smrtlink_5.0.1.9585.zip). The RNA-Seq data from 16 tissues of duck^23^ generated by our lab was used to correct nucleotide mismatches in consensus reads with the software LoRDEC^24^. Any redundancy in corrected consensus reads was removed by CD-Hit-Est^25^ to obtain final transcripts for the subsequent analysis. To estimate the completeness of our multiple tissue transcriptomic sequencing, we used a benchmarking universal single-copy orthologs (BUSCO) assessment^26^. We used ortholog sets from Aves lineages to examine transcriptome completion. We analyzed the completeness of datasets in processing steps, both corrected, polished consensus data and non-redundant transcript data.

### Functional annotation of PacBio isoforms

The obtained full-length transcripts were annotated by conducting a local BLASTx^27^ search against the protein databases, namely the Nr protein database at GenBank (http://www.ncbi.nlm.nih.gov), UniProtKB (http://www.expasy.ch/sprot, version:2019-8-14) and KOG. We determined the best match between each transcript and a known sequence based on the bit score. The results with a bit score below 50 were discarded and the highest bit score was considered as the best match. To classify the functions of transcripts based on molecular function, biological process and cellular component features, GO annotation was performed using Metascape^28^, while KEGG orthology and pathway annotations were obtained by using KAAS (KEGG Automatic Annotation Server)^29^. ANGLE^30^ was used to determine the open reading frame (ORF) of each full-length cDNA sequence. We used high confidence duck protein sequences (ftp://ftp.ensembl.org/pub/release-95/fasta/anas_platyrhynchos/cds/) for ANGLE training and then ran the ANGLE prediction for given sequences.

In addition to protein-coding RNAs, long non-coding RNAs constitute a major component of the transcriptome. In order to improve the accuracy of prediction of lncRNA, we used CPC (Coding Potential Calculator)^31^, PLEK (the predictor of long non-coding RNAs and messenger RNAs based on an improved k-mer scheme)^32^, Pfam-scan^33^ and CNCI (Coding-Non-Coding-Index)^34^ to predict the coding potential of transcripts after CD-Hit-Est, respectively. First, PLEK and CNCI were used to predict the coding potential according to the sequence characteristics of transcripts. The sequence of transcripts was compared with the known protein database by BLAST using CPC and searched by homology with Pfam-A and Pfam-B databases, their coding potential being predicted more accurately after comparing with the databases. The transcripts found by all programs were considered candidate lncRNA. Then, candidate lncRNA whose ORF length was longer than 300 bp and also had meaningful blast homology (BLASTX) when searched against the bird protein databases, were then removed. We determined the remaining non protein-coding transcripts as high confidence lncRNAs.

### Identification of AS modes

The full-length transcripts were mapped to the reference genome CAU_duck1.0 using GMAP^35^. The alignment file was filtered for 90% alignment coverage and 90% alignment identity and corresponding GFF files generated using cDNA_Cupcake^16^. SUPPA2^36^ generates the AS and transcript events from an annotation file (GFF/GTF format). It then generates two files: **ioe** format for local AS events, and **ioi** format for transcripts. The **ioe** file provides for each AS event in a gene and the transcripts that describe either form of the event. The **ioi** file provides for each transcript in a gene, the set of all transcripts from that gene from which the transcript relative abundance is calculated. The AS event generated by SUPPA2 contained five different types: Alternative 5′/3′ splice-site (A5/A3), Skipping exon (SE), Alternative first/last Exons (AF/AL), Mutually exclusive exons (MX) and Retained intron (RI).

## Data Records

The raw full-length data (Table 2) was deposited in the NCBI Sequence Read Archive (SRA) under accession number SRP188279^37^. The short-read RNA-Seq data used for correction was deposited in the SRA under accession number SRX3963450^38^, SRX3963443^39^, SRX3963442^40^, SRX3963441^41^, SRX3963440^42^, SRX3963439^43^, SRX3963438^44^, SRX3963437^45^, SRX3963436^46^, SRX3963435^47^, SRX3963434^48^, SRX3963433^49^, SRX3963432^50^, SRX3963431^51^, SRX3963429^52^, SRX3963428^53^. The full-length transcripts dataset generated from initial data were deposited in the NCBI Transcriptome Shotgun Assembly (TSA) database under accession number GHJL00000000.1^54^. The results of functional annotation and alternative splicing models were deposited in figshare^55^. The CAU_1.0 reference genome of duck was deposited in NCBI Assembly under accession number GCA_002743455.1^56^.

**Table 2:** Read number and length distribution after ISO-Seq analysis.

Subreads	Number	
Subreads number	14341324	
Average subreads length(bp)	2903	
N50(bp)	3259	
**Classify**		
CCS	702788	
5′-primer	605897	
3′-primer	653411	
Poly-A	641180	
Full length	563320	
Flnc	559454	
Average flnc read length(bp)	3338	
Consensus reads	313565	
**Correct**	**Before_correction**	**After_correction**
Total_number	313565	313565
Mean_length(bp)	3653	3698
Min_length(bp)	202	199
Max_length(bp)	15028	15233
N50(bp)	4017	4079
N90(bp)	2325	2341
**Cluster**	**Number of transcripts**	**Number of Genes**
<500 bp	311	169
500–1 kbp	985	541
1 k–2 kbp	29976	14409
2 k–3 kbp	92431	48198
>3 kbp	189862	136676
Total	313565	199993

## Technical Validation

### Quality control of sequencing analysis

From 77 Gb raw data, we produced 41.62 Gb subreads, which was classified into 702,788 non-chimeric circular consensus (CCS) reads. CCS reads comprised 563,320 full-length reads with an average read length of 3,338 bp. The 313,565 high-quality consensus isoforms and low-quality consensus isoforms were corrected with RNA-Seq data using LoRDEC. 199,993 corrected full-length isoforms were used for further analysis after accounting for redundancy (Table 2). We used Aves lineages (ortholog sets) to examine transcript completion (Table 3). As expected, the percentage of complete BUSCO genes is over 80% in full-length transcripts, both before and after removing redundancy. After the redundant sequences were removed, the complete duplicated sequence decreased by 12.4% and the number of complete single copy genes increased by 10.4%, indicating that the integrity of the full-length transcripts was not compromised by removal of the redundant sequences. Significantly reduced, non-redundant full-length transcript data sets showed high integrity for subsequent analysis.

**Table 3:** BUSCO analysis of transcript completeness.

BUSCO results	FL_after corrected		FL_NR	
Complete BUSCOs	4064	82.7%	3966	80.7%
Complete single-copy BUSCOs	1145	23.3%	1656	33.7%
Complete Duplicated BUSCOs	2919	59.4%	2310	47.00%
Fragmented BUSCOs	251	5.10%	320	6.50%
Missing BUSCOs	600	12.2%	629	12.80%
Total BUSCO groups searched	4915	100%	4915	100%

*FL: full-length.

### Annotation quality control

We annotated full-length transcripts with multiple reference databases for further study of gene function. First, the majority of transcripts (185,435; 92.72%) have similar sequences in Nt. Matches to other databases were as follows: 127,780 (63.89%) to Nr, 102,539 (51.27%) to UniProtKB and 53,570 (26.79%) transcripts aligned to the pfam database using BLASTx.

**Fig. 2:**
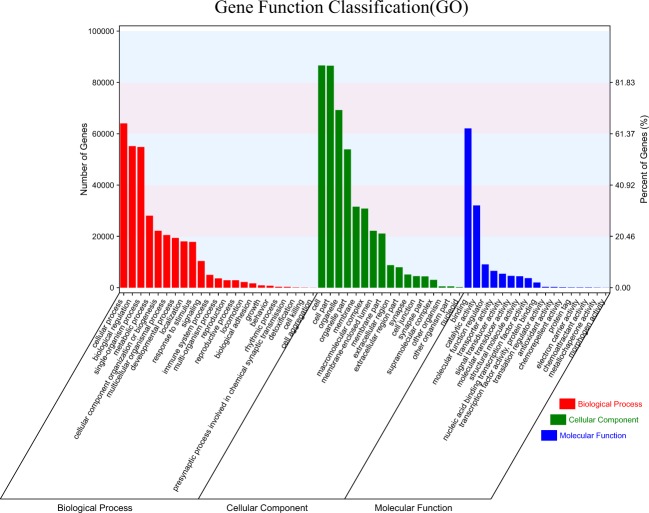
GO functional annotations of the Duck (*Anas platyrhynchos*) full-length transcripts. All GO annotations were classified into three categories according to ‘cellular components’, ‘biological processes’, and ‘molecular functions’. The X-axis shows gene functions. The number of transcripts with GO functions is indicated on the Y-axis.

All transcripts were subject to functional annotation and classification. About half of the full-length transcripts were annotated by KEGG, GO and KOG databases. In general, 187,139 (93.57%) transcripts were found in at least one database and 20.81% of the transcripts were found in all databases (Table 4). The metascape website first obtained GO annotations from Gene Ontology (http://geneontology.org/, 2019-07-01)^57^. GO terms were assigned to each isoform based on the corresponding homologs in UniProtKB database. A total of 97,823 (48.91%) transcripts were annotated to multiple GO classification terms. In the “biological process” category, the majority of the transcripts were represented by ‘cellular process’ (63,996), ‘biological regulation’ (55,142) and ‘single-organism process’ (54,805) terms. On the other hand, ‘cell’ (86,603) was the most represented item in the “cellular component” category, while ‘binding’ (62,070) was the most common term in the “molecular function” category (Fig. 2). Further analysis of the KEGG annotations revealed that most transcripts were enriched in signal transduction (13,791), endocrine system (6,935), immune system (5,791), cellular community-eukaryotes (5,744) and transport and catabolism (5,645). With KOG analysis, 82,456 (41.23%) transcripts were annotated and classified into 26 KOG categories. The largest cluster was “Signal transduction mechanisms (T)”, indicating that most of the function represented by these transcripts are for the basic mechanisms controlling cell growth, proliferation, metabolism, and many other processes. The next largest cluster was ‘the general function prediction only (R)’, followed by ‘Posttranslational modification, protein turnover, chaperones (O)’, ‘Cytoskeleton (Z)’ and ‘Transcription (K)’.

**Table 4:** Annotation statistics.

Database	Full-length transcripts of Duck
UniProtKB	102539 (51.27%)
Nr	127780 (63.89%)
Nt	185435 (92.72%)
KOG	82456 (41.23%)
GO	97823 (48.91%)
KEGG	116503 (58.25%)
Pfam	53570 (26.79%)
At least one database	187139 (93.57%)
All database	41614 (20.81%)

**Fig. 3:**
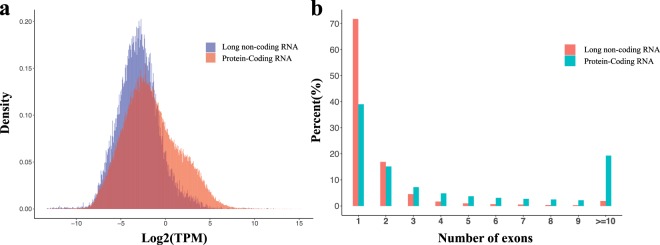
Characterization of identified novel lncRNAs. (**a**) Comparison of isoform expression between lncRNA and protein-coding RNA data. (**b**) The Number of exons in lncRNAs and protein-coding RNAs. In general, the number of exons in protein-coding RNAs is higher than in lncRNAs. More than 70% of the lncRNAs are represented by single-exon transcripts.

We obtained 34,364 candidate lncRNAs determined by the coding ability of the predicted sequence. In order to improve the accuracy of predicted lncRNA, sequences with ORF > 300 bp and which aligned against the avian protein databases were excluded, leaving 23,755 remaining sequences. The average gene expression of predicted lncRNAs is much lower than that of protein-coding RNAs (Fig. 3). In addition, the number of exons in lncRNAs is also significantly less than that of protein-coding RNAs. 71.72% of the predicted lncRNAs have only a single exon and only 11.36% of lncRNAs have more than two exons (Fig. 3).

### Quality control of AS events

**Fig. 4:**
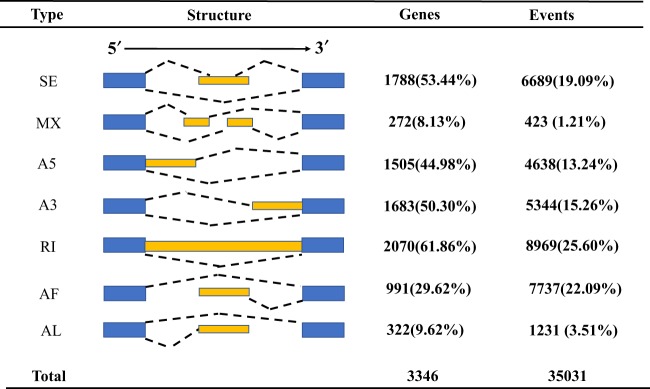
The total number of AS events in detected genes and transcripts by SUPPA2 analysis. A3, alternative 3′ splice site; SE, skipped exon; A5, alternative 5′ splice site; AF, alternative first exon; MX, mutually exclusive exon; AL, alternative last exon; RI, retained intron.

**Fig. 5:**
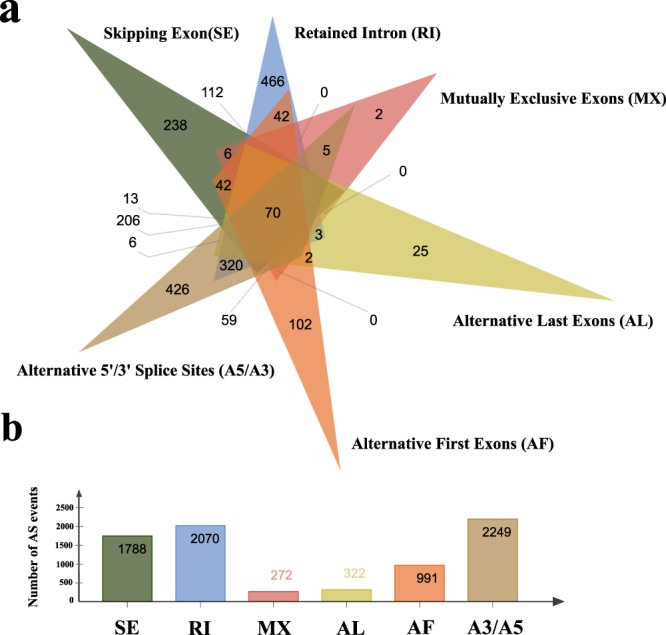
Distribution of alternative splicing events in Genes. (**a**) Overlap of the different types of AS mechanisms; (**b**) The number of genes that identified different types of alternative splicing.

More than 99% of full-length transcripts were mapped to the reference genome, and 18,328 gene models predicted (Table 5). We identified 35,031 AS events from 3,346 gene models. RI predominated, accounting for 61.86% of alternative transcripts. Except for AL (9.62%) and MX (8.13%), other AS types, such as RI (61.86%), SE (53.44%), A3 (50.30%), A5 (44.98%) and AF (29.63%), are more common in alternative splicing events (Fig. 4). Most genes exhibited only one model of AS, with only 70 genes showing every AS type (Fig. 5). We found that the number of AS events within genes is correlated with the number of exons, indicating that the complexity and diversity of transcription is enhanced by AS as exons increase.

**Table 5:** Alignment statistics for full-length transcripts after correction with Illumina data.

Alignment results	All full-length transcripts	Percent (%)
Unaligned	1719	0.86%
Multi-mapped	10488	5.24%
Uniquely Mapped	187786	93.90%
qCoverage = 100%	61745	30.87%
qCoverage > =99%:	53135	26.57%
qCoverage > =90%	34568	17.28%
Total number transcripts	199993	100.00%

The data provided in this study form the first report of a full-length transcriptomic resource for ducks, which includes predicted lncRNA and AS events identified by Iso-seq technology. These findings will be invaluable for improving genome annotation, examining AS evolution, and conducting functional studies in ducks.

## Data Availability

Most of the data analysis was completed by software running on the Linux system, and the version and parameters of main software tools are described below. (1) SMRTlink: version 5.1, parameters: no_polish TRUE, max_drop_fraction 0.8, min_zscore −9999.0, min_length 50, min_predicted_accuracy 0.8, max_length 15000, min_passes 2. (2) Arrow: parameters: bin_size_kb 1, hq_quiver_min_accuracy 0.99, qv_trim_5p 100, qv_trim_3p 30, bin_by_primer false. (3) LoRDEC: version V0.7, parameters: -k 23, -s 3. (4) CD-Hit-Est: version 4.6, parameters: -c 0.95 -T 6 -G 0 - aL 0.00 -aS 0.99. (5) BUSCO: version 3.0.2, default parameters. (6) Blastx: version 2.2.31, parameters: -outfmt 6, e value:1e-5, -num_descriptions 10, -line_length = 60. (7) CNCI: version 2, default parameters. (8) CPC: version 0.9, parameters: 1e-10. (9) Pfam-scan:31.0, parameters: -E 0.001 –domE 0.001. (10) PLEK: version 1.2, parameters: -minlength 200. (11) GMAP: version gmap.sse42, parameters: -f samse -n 0 -z sense_force -t 8. (11) SUPPA2: version 2.2.1, default parameters.
